# Toward the analysis of functional proteoforms using mass spectrometry-based stability proteomics

**DOI:** 10.3389/frans.2023.1186623

**Published:** 2023-06-21

**Authors:** Ji Kang, Meena Seshadri, Kellye A. Cupp-Sutton, Si Wu

**Affiliations:** Department of Chemistry and Biochemistry, University of Oklahoma, Norman, OK, United States

**Keywords:** proteomics, protein thermal stability, proteoform, mass spec, top-down proteomics

## Abstract

Functional proteomics aims to elucidate biological functions, mechanisms, and pathways of proteins and proteoforms at the molecular level to examine complex cellular systems and disease states. A series of stability proteomics methods have been developed to examine protein functionality by measuring the resistance of a protein to chemical or thermal denaturation or proteolysis. These methods can be applied to measure the thermal stability of thousands of proteins in complex biological samples such as cell lysate, intact cells, tissues, and other biological fluids to measure proteome stability. Stability proteomics methods have been popularly applied to observe stability shifts upon ligand binding for drug target identification. More recently, these methods have been applied to characterize the effect of structural changes in proteins such as those caused by post-translational modifications (PTMs) and mutations, which can affect protein structures or interactions and diversify protein functions. Here, we discussed the current application of a suite of stability proteomics methods, including thermal proteome profiling (TPP), stability of proteomics from rates of oxidation (SPROX), and limited proteolysis (LiP) methods, to observe PTM-induced structural changes on protein stability. We also discuss future perspectives highlighting the integration of top-down mass spectrometry and stability proteomics methods to characterize intact proteoform stability and understand the function of variable protein modifications.

## Introduction

Elucidating protein functionality is very important for understanding biological systems. The study of protein structure, function, and interactions has traditionally been done using classic structural biology methods, including protein nuclear magnetic resonance (NMR)(Yee et al, 2002), protein crystallography (Smyth and Martin, 2000), and cryogenic electron microscopy (cryo-EM)(Nickell et al, 2006). These methods are widely used, have high structural resolution, and can inform protein structural conformation as well as characterize interactions. Protein NMR has typically been applied for proteins from 10–30 kDa; however, some methods for protein NMR can be applied to very large proteins (>100 kDa) (Hu et al, 2021). Protein NMR can also assess protein structure and protein dynamics in solution and native-like environments. X-ray crystallography is the most common method for protein structural determination accounting for nearly 90% of structures in the PDB database (Benjin and Ling, 2020). More recent advances to Cryo-EM technology has allowed atomic resolution and made its use for protein structural determination more common (Yip et al, 2020).

However, despite these advantages, protein NMR and crystallography suffer from requirements for a relatively large amount of pure protein; time consuming protein expression and purification are often required. For X-ray crystallography, protein purification is followed by the need to grow protein crystals which can be challenging, particularly for disordered proteins and membrane proteins. Crystalline proteins may also have conformations that are not consistent with proteins in solution (Swaminathan et al, 2003; Acharya and Lloyd, 2005). Protein NMR often requires proteins to be expressed with heavy isotopes, uses solutions with high concentration (mM), and data analysis can be challenging and time-consuming (Venien-Bryan et al, 2016). Cryo-EM is currently unable to produce high resolution structures for proteins smaller than 30–50 kDa (D’Imprima and Kühlbrandt, 2021). Additionally, proteoforms with functional modifications such as post-translational modifications (PTMs) relevant to protein function generally cannot be expressed and purified as required for traditional structural biology approaches.

Overall, these structural biology methods for determination of protein function are robust and high resolution; however, drawbacks including low-throughput analysis of single proteins, requirement for large amounts of pure protein, and inability to characterize protein modifications has encouraged exploration into alternative methods for protein structural analysis. Hence, methods that provide structural and functional analysis of proteins using mass-spectrometry (MS)-based proteomics have been developed (Aebersold and Mann, 2003; Aebersold and Mann, 2016). While the resolution of these MS-based proteomics methods tends to be lower than the traditional structural biology methods discussed above, they benefit from high sensitivity, ability to analyze low purity samples or complex samples, and high throughput. Of particular interest is the ability of the MS-based methods to observe the structure and function of modified proteoforms (*e.g.*, amino acid substitutions and post-translational modifications) that are often functionally relevant. MS-based proteomics methods capable of analyzing the structure, functions, and interactions of modified proteoforms include protein footprinting and stability proteomics methods.

A suite of MS-based protein footprinting methods, including Hydrogen-Deuterium Exchange mass spectrometry (HDX-MS) (Konermann et al, 2011), Fast Photochemical Oxidation of Protein (FPOP) (Johnson et al, 2019), Hydroxyl Radical Footprinting (HRF) (Xu et al, 2003), and Flash OXidation (FOX) (Sharp et al, 2021) have been developed to characterize functional proteins as well as their structures. Protein footprinting methods generally chemically label specific amino acid residues (or, in the case of HDX-MS, the amide backbone) that are solvent accessible in their native state to probe the protein composition, structure, and binding (Liu et al, 2020). Footprinting methods are generally low resolution when compared with the traditional structural biology methods discussed previously, typically achieving peptide-level resolution for HDX-MS analysis. Resolution for covalent labeling footprinting methods (excluding HDX-MS) can be limited by the existence of amino acid residues or functional groups appropriate for labeling. Initially, these protein footprinting methods were limited to pure protein and simple protein mixtures (Takamoto and Chance, 2006; Jones et al, 2011; Zhang et al, 2011; Sharp et al, 2021); however, more recent platforms such as those utilizing low-temperature LC separation for HDX-MS(Fang et al, 2021a) and more advanced data analysis techniques (Rinas and Jones, 2015) or in cell labeling (Kaur et al, 2020) for FPOP have enabled analysis of complex protein mixtures.

A series of MS-based proteomics methods, ‘stability proteomics’ has been developed to elucidate functional changes of proteins in complex mixtures (Kaur et al, 2018; Le Sueur et al, 2022). The tertiary structure of a protein is stabilized by various interactions within the proteins, including hydrophobic interactions, electrostatic interactions, hydrogen bonding, ionic bonding, and covalent bonding (disulfide bonds) (Gromiha, 2010; Pace et al, 2014). Stability proteomics generally falls into two categories: proteolysis-based methods and denaturation-based methods. Proteolysis-based methods, including Drug Affinity Responsive Target Stability (DARTS) (Lomenick et al, 2009) and Limited Proteolysis coupled with Mass Spectrometry (LiP-MS) (Feng et al, 2014), utilize the resistance of a protein to proteolysis to probe protein stability. Denaturation-based methods probe protein stability by studying the tendency of a protein to denature when exposed to a denaturing (chemical or thermal) gradient (Kaur et al, 2018). A few commonly applied denaturation-based methods include Stability of Proteins from Rates of Oxidation (SPROX) (West et al, 2008), Thermal Proteome Profiling (TPP) (Savitski et al, 2014), Chemical denaturation and Protein Precipitation (CPP) (Meng et al, 2018), Ion-based protein precipitation with Proteome-Integrated Solubility Alteration (i-PISA) (Beusch et al, 2022), and pulse proteolysis (Park and Marqusee, 2005; Chang et al, 2012).

These methods utilize the resistance of a protein to chemical or thermal denaturation or proteolysis to gauge protein stability. Protein stability can be altered by ligand binding (Celej et al, 2003; Majewski et al, 2019), protein-protein interactions (Layton and Hellinga, 2011), and protein modification (Le Sueur et al, 2022); as such, change in protein stability can be indicative of protein function (Teilum et al, 2011). The advantage of stability proteomics is the ability to analyze protein stability in complex protein mixtures (i.e., native or native-like environments) to observe proteome-level changes in protein stability. These methods have been used to observe the effect of ligand binding for drug target elucidation as well as to observe binding to non-target proteins, the effect of mutation and post-translational modification on proteome stability, and the effect of disease on proteome stability among other applications. Combinations of these techniques have also been evaluated to compare and contrast identified drug binding proteins between methods and compare proteome coverage (Cabrera et al, 2020; Xu et al, 2021). While these methods may offer limited (Strickland et al, 2013) or no resolution for the site of protein interactions, they provide a unique look at the functional proteome to observe unknown interactions and changes in protein stability.

As protein modifications due to alternative splicing, amino acid substitution, and post-translational modification are relevant to protein function by way of PTM crosstalk, protein-protein interactions, and protein activity (e.g., PTM switching (Ansong et al, 2013)), the application of these MS-based proteomics methods to modified functional proteoforms is of particular interest to researchers. In this review, we discuss the application of these stability proteomics methods (Supplementary Table S1), with a focus on TPP, to functionally modified and mutated proteoforms as well as the future perspectives regarding the application of stability proteomics methods to top-down proteomics.

### Thermal proteome profiling

Among currently available stability proteomics methods, TPP which is based on the principles of the cellular thermal shift assay (CETSA) (Molina et al, 2013) is the most widely used to profile proteome-wide protein thermal stability of complex protein mixtures or intact cells (Savitski et al, 2014; Mateus et al, 2018; Mateus et al, 2020a). In a typical TPP experiment, aliquots of a sample (cells, cell lysate, tissue, etc.) are incubated at increasing temperatures, causing proteins to denature and precipitate at a specific temperature (melting point, T_m_). Precipitates are removed and the soluble protein fractions are analyzed using bottom-up mass spectrometry with quantitative isobaric mass tag labeling and data dependent acquisition (Franken et al, 2015; Zhang et al, 2020) or label-free quantitation with data independent acquisition (George et al, 2023). Normalized protein abundance is plotted against the temperature, and the melting curve for each protein is fit using diverse methods/software packages that can determine melting point shifts and melting behavior similarities (Savitski et al, 2014; Leuenberger et al, 2017; Childs et al, 2019; Mateus et al, 2020a; Kurzawa et al, 2020; Fang et al, 2021b). Thermal proteome profiling has been used to study the melting behavior of proteins *in situ* under differing cellular conditions (Leuenberger et al, 2017; Mateus et al, 2020a).

TPP methods have been expanded and adapted for various purposes including drug target discovery and determination of binding strength. These methods include one-dimensional assays that utilize thermal gradients, concentration gradients, or isothermal dose-response (Franken et al, 2015; Lim et al, 2018). TPP methods have also been applied to many types of sample including intact cells, tissue, and whole blood (Perrin et al, 2020; Kalxdorf et al, 2021; Ruan et al, 2022; Zhao et al, 2022). Multidimensional methods that use both thermal and concentration gradients have also been developed to investigate thermal stability and binding affinity simultaneously (Becher et al, 2016; Mateus et al, 2018; Dziekan et al, 2020). Additionally, methods to probe the effect of protein expression, such as gene deletion in mutant strains (Mateus et al, 2020b), on protein expression and stability of the entire proteome have been developed. These are just a few of the interesting applications of TPP methods; others have been extensively reviewed in the literature (Mateus et al, 2017; Mateus et al, 2020a; de Souza and Picotti, 2020; Le Sueur et al, 2022; Mateus et al, 2022).

**FIGURE 1:**
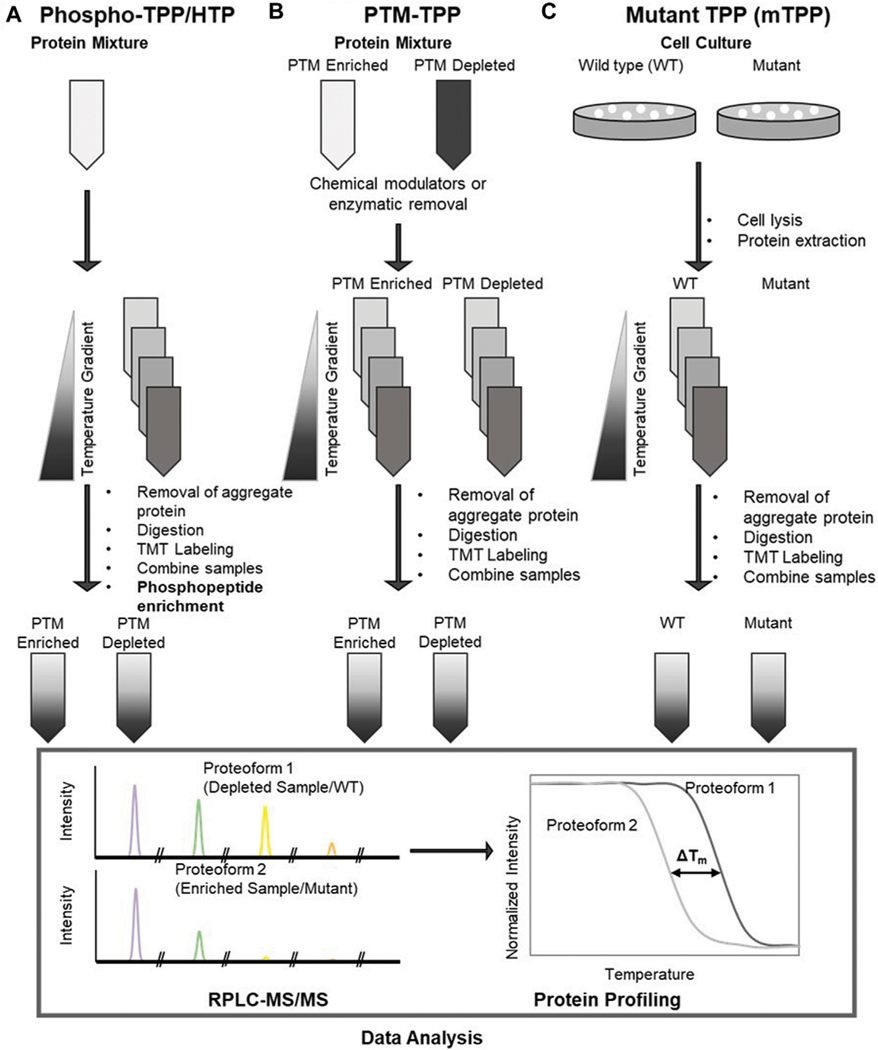
Workflows for **(A)** phospho-TPP/hotspot thermal profiling (HTP), [schematic derived from Huang, J. X., et al. (2019)]; **(B)** post translational modification TPP (PTM-TPP) [schematic derived from King, D. T., et al. (2022)]; **(C)** mutant TPP (mTPP) [schematic derived from Peck Justice, S. A., et al. (2020)].

Recently, methods such as Hotspot Thermal Proteome Profiling (HTP) (Huang et al, 2019), Post-Translational Modification Thermal Proteome Profiling (PTM-TPP) (King et al, 2022), and Mutant Thermal Proteome Profiling (mTPP) (Peck Justice et al, 2020) were developed to assess changes in protein function due to structural changes such as variable PTMs and mutation (Figure 1). Similarly, System-wide Identification and prioritization of Enzyme Substrates by Thermal Analysis (SIESTA) was developed to identify and characterize enzyme substrates by observing the effect of enzyme catalyzed PTMs on substrate stability. (Saei et al, 2021). PTMs are covalent modifications of proteins that can alter protein function and increase the functional diversity of the proteome (Bagwan et al, 2021). Functionally relevant PTMs can affect protein function in a variety of ways; for example, modulation of protein-protein interactions (PPIs) (Seet et al, 2006; Duan and Walther, 2015), ATP production (Ardito et al, 2017), and function of cell surface and extracellular proteins in cancer and autoimmune disease (Reily et al, 2019). As such, high-throughput examination of PTM functionality has been a focus for advancement of the TPP methods discussed here.

A subsect of TPP techniques, phospho-TPP (Potel et al, 2021; Smith et al, 2021) and HTP (Huang et al, 2019), have been developed to examine the effects of endogenous phosphorylation on thermal stability and protein functionality. Phosphorylation is of particular interest because it is an extremely common transient PTM that influences protein binding, stability, and dynamics (Johnson, 2009; Nishi et al, 2014). Combination of phosphopeptide enrichment strategies and TPP was first introduced by Azimi et al to determine the effect of protein phosphorylation on thermal stability of the phosphoproteome of SK-Mel 24 and SK-Mel 28 human carcinoma cells, which provided insights on protein interactions that could be targeted to counteract drug resistance of SK-Mel 28 (Azimi et al, 2018). This study demonstrated that these phospho-TPP methods enable determination of the effect of phosphorylation on thermal stability and protein function.

While analysis of the stability of HEK293T proteome by Huang et al did not report a significant difference in the T_m_ of the proteome and phosphoproteome, significant changes in the melting behavior for 25% of phosphorylated sites were reported (Huang et al, 2019). However, independent studies have shown that only 1.5%–3% of phosphosites significantly impact thermal protein stability (Potel et al, 2021; Lenz and Stühler, 2022). An improved HTP workflow involving high-pH reverse phase fractionation prior to phosphopeptide enrichment, label-fractionate-enrich (LFE)-HTP, has been reported for deeper phosphoproteome coverage (Stein et al, 2021).

TPP methods have also been applied to study the effect of PTMs such as O-GlcNAcylation on thermal stability (King et al, 2022). To analyze stability differences between modified proteoforms, two methods were used to observe the effect of O-GlcNAc on protein stability: enzymatic removal TPP (erPTM-TPP) and chemical modulators TPP (cmPTM-TPP). Though O-GlcNAc has often been reported to stabilize proteins (Drake et al, 2018), PTM-TPP methods found that O-GlcNAc tends to destabilize proteins due to interference in protein-protein interactions and its competition with protein phosphorylation (Issad et al, 2022).

Mutations of amino acids can disrupt protein function and cause disease by disrupting protein interactions (Zhong et al, 2009; Sahni et al, 2015), prevention of catalysis, allosteric regulation, disruption of a PTM, or, most commonly, changing the protein folding structure/stability (Wang and Moult, 2001; Bromberg and Rost, 2009). Additionally, site-directed mutagenesis is useful for interrupting standard gene activity and functional studies. As such, mutant TPP (mTPP) was developed as a high-throughput method to study the direct causes/pathways for genetic disorders and effects of site-directed mutagenesis and observe proteome-wide shifts in thermal stability resulting from amino acid substitution. mTPP was first introduced by Peck Justice et al (Peck Justice et al, 2020) to study temperature-sensitive (TS) mutants, specifically how changes in protein structure affected the thermal stability of the TS mutants compared to the unmodified protein and the PPI network as a whole. In a further application of mTPP by the Mosely group, (Peck Justice et al, 2021) applied affinity-purified protein complex as an isobaric trigger channel to determine the melting points of low abundance proteins.

Vieitez et al studied the functional relevance of phosphorylation on 474 phospho-deficient yeast strains (Viéitez et al, 2019). Screening their fitness in 102 different conditions showed changes in thermal stability in 8 phospho-mutants, with one exhibiting an increase and another a decrease in stability that was not statistically significant. (Banzhaf et al, 2020). studied the effect of an NlPI mutant on lipoproteins, finding that the deletion of NlPI altered protein abundance and thermal stability. While mTPP helps to understand the effect of mutations on protein stability, producing the TS mutants used in these studies often results in many mutations, making it difficult to determine which mutations cause which changes in protein stability. (Peck Justice et al, 2020).

In another interesting application of TPP, SIESTA utilizes the effect that PTMs have on protein thermal stability to identify known and unknown enzyme substrates (Saei et al, 2021). In SIESTA, lysate is incubated with an enzyme of interest and the enzyme cosubstrate to facilitate enzymatic post-translational modification of enzyme substrates. These modified substrates often demonstrate altered thermal stability resulting in an unbiased and high throughput method to identify enzyme substrates.

Overall, these methods depend on enrichment or overexpression of protein modifications to determine the effect of PTMs on protein stability. This is generally required as modified proteins tend to be lower in abundance than their modified proteoforms. However, very recently, (Kurzawa et al, 2023), introduced an alternative approach that applied a deep peptide identification method to TPP to observe the effect of modification on the thermal stability of coexisting functional proteoform groups. This technique did not require enrichment or overexpression of modified peptides and allowed more general analysis of changes in thermal stability caused by modifications such as PTMs, PPIs, etc.

### Other stability proteomics methods for analysis of PTM induced stability shifts

While TPP is the most widely used stability proteomics technique, particularly for examining the effects of PTMs on protein stability, other stability proteomics methods, including SPROX and LiP, have been applied to this end. SPROX is unique in that it utilizes covalent labeling (e.g., methionine oxidation) to monitor protein unfolding (West et al, 2008). Proteins are unfolded using a chemical denaturant gradient and exposed to oxidizing conditions via the addition of hydrogen peroxide (H_2_O_2_) to selectively oxidize exposed methionine residues under each denaturant condition (Strickland et al, 2013). Oxidation is followed by a quenching agent (e.g., free methionine) to remove excess H_2_O_2_ and control labeling time. Proteins are subjected to digestion and methionine containing peptides are quantified using TMT labeling methods to determine the concentration at which proteins unfold and methionine residues become oxidized. SPROX also allows the examination of site-specific unfolding to determine the location of conformational changes or protein interactions.

LiP is another stability proteomics method that utilizes the resistance of a protein to proteolysis to determine protein stability (Feng et al, 2014). In LiP, proteins are digested with a nonspecific protease in the native state for a particular amount of time (Malinovska et al, 2023). Under these conditions, proteins with more accessible or unstructured regions tend to undergo more proteolysis than proteins with more stable structures. This reaction is quenched by heating and chemical denaturation, and the samples are further digested with a secondary protease such as trypsin. Tryptic and semitryptic/half-tryptic peptides are quantified using label-free MS-based methods to determine the extent to which a protein was digested in the native state.

**FIGURE 2:**
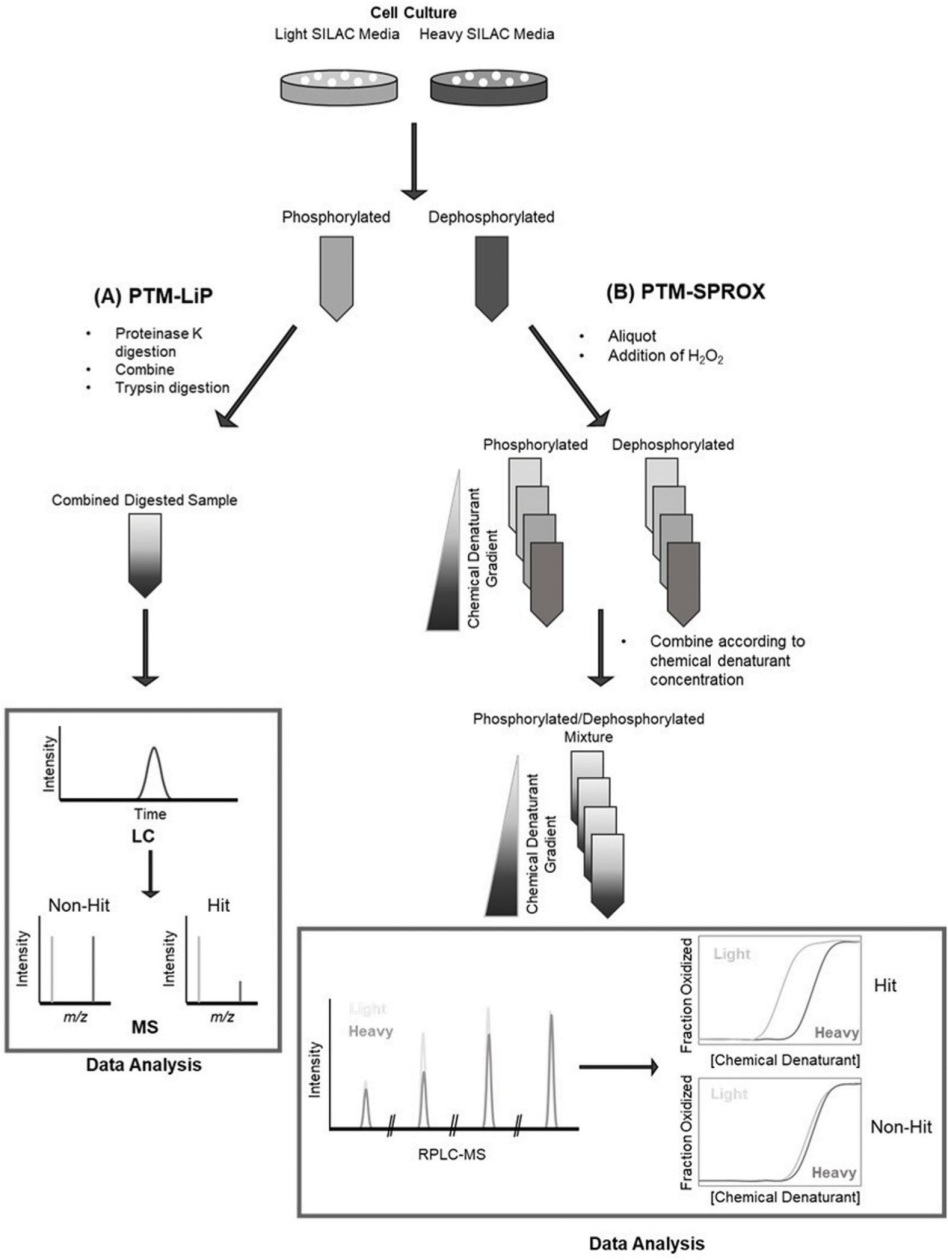
Workflows for **(A)** post translational modification-limited proteolysis (PTM-LiP); **(B)** post translational modification stability of proteins from rates of oxidation (PTM-SPROX), [schematics derived from Meng, H, et al. (2018)].

SPROX and LiP have been applied to the analysis of the effect of phosphorylation on proteoform stability to study the significance of phosphorylation events in the MCF-7 breast cancer cell line, Figure 2 (Meng and Fitzgerald, 2018). MCF-7 cells were cultured in heavy and light SILAC media and the heavy lysate was treated with alkaline phosphatase to remove protein phosphorylation. These samples were subjected to LiP or SPROX workflows to determine changes in protein stability as a function of phosphorylation. Overall, SPROX and LiP found hundreds of proteins with changes in stability related to dephosphorylation. Additionally, these methods were able to identify changes in stability of proteins known to be differentially phosphorylated or stabilized in human breast cancer and proteins that are known to have function modulated by phosphorylation (Cappelletti et al, 2021) also applied LiP to the study of overall proteome stability in response to stress or nutrient adaptation. This study was also able to observe changes in protein stability due to phosphorylation in response to osmotic stress (Cappelletti et al, 2021).

While LiP and SPROX are the only stability proteomics methods, apart from TPP, that have been applied to the analysis of the effects of PTMs on protein stability, other methods such as pulse proteolysis could also potentially be applied for this purpose. Pulse proteolysis is similar to SPROX in that it probes protein stability using a chemical denaturation gradient (Park and Marqusee, 2005; Chang et al, 2012). Chemical denaturation is followed by a short proteolysis to digest denatured proteins.

### Perspectives and future applications

Stability proteomics methods have seen an astonishing number of modifications and developments to make these techniques extremely versatile and applicable for a wide variety of purposes. The stability proteomics methods and applications reviewed here are unique in their goal of examining the effect of protein structural changes such as PTMs and mutation on protein stability or interaction. These methods have been applied with much success to determine the changes in stability caused by these modifications on the affected proteins directly, on protein interactions, and on the proteome generally. The bottom-up proteomics techniques currently applied to the stability proteomics methods reviewed here are robust, high-resolution, and have low limits of detection; however, the required proteolysis inhibits the examination of how structural changes affect intact proteoform stability (Catherman et al, 2014). As many PTMs of analogous and differing varieties can exist simultaneously on an intact proteoform to effect protein function and interactions, as is concisely demonstrated by the histone code (Jenuwein and Allis, 2001; Fornelli et al, 2018), protein digestion makes characterization of the intact, biologically active proteoform challenging. Furthermore, implementation of PTM specific enrichment strategies at the peptide level, as is done in many of the methods reviewed here, restricts the ability of these methods to observe how different numbers of PTM instances and combinations of different types of PTMs effect the stability of these proteoforms. As modified proteoforms can not only change the function of proteins but can also be the cause of or demonstrative of disease states, analysis of the total PTM profile of proteins is critical for biochemical analysis. As such, observation of the intact modified proteoforms directly can give a better understanding of how functional proteoforms perform and interact *in vivo*.

In contrast to bottom-up approaches, top-down proteomics approaches analyze intact proteins directly so that the structure of functional proteoforms can be examined (Gregorich and Ge, 2014). While there are several limitations to application of high-throughput top-down proteomics, a primary disadvantage is the lack of methods to assign functional annotations to intact proteoforms as recently reviewed by (Melby et al, 2021) This challenge stems from fundamental disadvantages in top-down proteomics including relatively low sensitivity hindering characterization of low abundance proteoforms, limitations in the size of proteoforms that can be analyzed using high resolution MS methods, and difficulty in applying quantitative proteomics methods to intact proteins. With these limitations, application of methods designed to probe protein functionality, such as stability proteomics, is very challenging using top-down methods. Fortunately, recent advancements in the area of top-down proteomics would allow application of some of these stability proteomics methods to examine how protein stability relates to unique protein proteoforms resulting in more detailed information regarding how protein modifications and combinations of modifications are involved in regulating protein function.

Intact proteoforms have many possible charge states resulting in wide charge state envelopes that decrease overall sensitivity. For high-throughput top-down proteomics, the effects of decreased sensitivity are made more prominent when proteins are coeluted from online separation methods which can result in ion suppression and difficulties in characterization, particularly of low abundance proteoforms (François et al, 2009; Pirok et al, 2019). Methods such as parallel ion parking (Chrisman et al, 2006) and ion/ion proton transfer (Stephenson and McLuckey, 1996) have been developed to narrow the charge envelope and increase sensitivity for MS analysis, and gas phase separation methods such as FAIMS have been implemented to decrease chemical noise and ion suppression (Gerbasi et al, 2021; Kaulich et al, 2022). Improving separation to decrease spectral complexity has also been implemented to increase depth of proteome analysis and improve intact proteoform characterization. In this vein, multidimensional separation is a technique that utilizes sequential, orthogonal separation techniques to improve separation resolution, proteome coverage, and characterization of proteins in a wide dynamic range. (Melby et al, 2021). However, the primary quantitative proteomics technique currently applied to top-down proteomics is label-free quantitation (Cupp-Sutton and Wu, 2020). As label-free quantitation requires an individual analysis for each experimental condition, it is not compatible with multidimensional separation. As such, application of other quantitative proteomics methods to top-down proteomics will improve quantitative accuracy and throughput.

Apart from label-free quantitation, the quantitative proteomics techniques currently applicable to top-down proteomics are metabolic labeling techniques and isobaric chemical tag labeling. (Cupp-Sutton and Wu, 2020). These techniques benefit from being compatible with multidimensional separation as well as having multiplex options to improve proteomic throughput. While metabolic labeling techniques have been applied for top-down proteomics, they must be implemented during cell growth to incorporate isotopic variants of amino acids into the protein primary structure (Ong et al, 2002). Therefore, metabolic labeling techniques are limited to samples that can be grown in lab and are not commonly used in stability proteomics methods. Isobaric chemical tag labeling, however, can be applied to any protein sample for flexible application. Until recently, isobaric chemical tag labeling techniques have not been widely applicable to high-throughput top-down proteomics due to issues with protein solubility and improper labeling under standard labeling conditions (Hung and Tholey, 2012). Of late, optimization of TMT labeling techniques including sample preparation and fragmentation have been developed for complex mixtures of intact proteins (Winkels et al, 2021; Yu et al, 2021; Guo et al, 2022a; Guo et al, 2022b; Guo et al, 2023). Application of these novel methods would allow for highly multiplexed TDP and application of multidimensional separations for improved quantitation and depth of proteome characterization.

Overall, developments in top-down proteomics including improved instrumental analysis, sample separation, and quantitation as discussed here have paved the way for application of top-down proteomics for more complex experimental designs such as stability proteomics methods. Stability proteomics techniques that do not require proteolysis (e.g., LiP, pulse proteolysis, DARTS) may be compatible with top-down proteomics, such as TPP, SPROX, CPP, PISA, and i-PISA. For example, TPP utilizes a thermal denaturation gradient to probe protein stability; therefore, the supernatant containing the soluble proteins can be directly analyzed using quantitative top-down proteomics approaches. MS-compatible buffers or online desalting techniques can be used to directly analyze TPP samples without additional sample preparation steps. Recently, the Wu lab has developed a label-free top-down TPP platform that has been applied to plot thermal profiles for intact proteoforms in complex samples such as *Escherichia coli* and human cell lysate (unpublished results). One possible challenge in analyzing these samples is the incomplete removal of precipitated proteins, which may cause column clogging, and improved methods for aggregate protein removal may be required. Applying top-down isobaric chemical tag labeling techniques can also improve the throughput of top-down TPP applications (Winkels et al, 2021; Yu et al, 2021; Guo et al, 2022a; Guo et al, 2022b; Guo et al, 2023).

Other methods such as SPROX, CPP, and i-PISA can also be coupled with top-down proteomics methods. However, these techniques use high concentrations of MS-incompatible buffers and reagents during sample preparation that can interfere with ion generation and lead to ion suppression during ESI and MS detection. For example, SPROX and CPP utilize high concentrations of chemical denaturants such as urea or guanidinium chloride to destabilize proteins, and i-PISA uses copper chloride for kosmotropic protein precipitation. Coupling existing methods (online or offline) for desalting intact protein samples (Cai et al, 2016) or protein precipitation and resolubilization [e.g., chloroform-methanol-water precipitation (Doucette et al, 2014)] would allow top-down proteomics analysis for these stability proteomics methods. These additional sample handling steps can lead to sample loss and increased error which decreases quantitative accuracy, particularly for label-free quantitation. Application of the intact protein isobaric chemical tag labeling protocol discussed above would necessitate buffer exchange prior to labeling regardless of MS compatibility of reagents used, and multiplexing limits run-to-run variability issues and reduces error to improve quantitation accuracy. Apart from these considerations, the application of top-down MS methods to stability proteomics methods largely overlap with bottom-up methods as demonstrated in Supplementary Table S1 including the resistance of proteins to certain types of denaturation and complexity in the denaturation behavior. To address some of these drawbacks, multiple proteomics methods have been applied to improve target identification and cross-verify identified targets (Cabrera et al, 2020; Bailey et al, 2023).

Overall, coupling top-down proteomics with the stability proteomics platforms reviewed here that focus on the characterization of the effect of protein modification on structural stability will open up the area of top-down proteomics for functional annotation of proteoforms, an area of research previously challenging to achieve.

## Supplementary Material

supplementary table 1

## Data Availability

The original contributions presented in the study are included in the article/Supplementary Material, further inquiries can be directed to the corresponding author.
