# Supplementary material for: Toward the analysis of functional proteoforms using mass spectrometry-based stability proteomics
Source: Front Anal Sci. Author manuscript; Available in PMC 2024 Jul 26. (PMC11281393; doi:10.3389/frans.2023.1186623)
Supplement: supplementary table 1 [file NIHMS1925944-supplement-supplementary_table_1.docx]

Toward the Analysis of Functional Proteoforms using Mass Spectrometry-based Stability Proteomics

Ji Kang^1^, Meena Seshadri^1^, Kellye A Cupp-Sutton^1*^, Si Wu^1*^

^1^Department of Chemistry and Biochemistry, University of Oklahoma, Norman, OK 73019

***Correspondence:**

Si Wu, Ph.D., Department of Chemistry and Biochemistry, 101 Stephenson Parkway, Room 2210, Norman, Oklahoma 73019-5251

Email: si.wu@ou.edu

Fax: (405) 325-6111

or

Kellye A Cupp-Sutton, Ph.D., Department of Chemistry and Biochemistry, 101 Stephenson Parkway, Room 3550, Norman, Oklahoma 73019-5251

Email: Kellyecuppsutton@ou.edu

Keywords: (Min.5-Max. 8)

Proteomics, protein thermostability, proteoform, mass spectrometry; top-down proteomics; post-translational modification

**Supplementary Table 1. Advantages and limitations of stability proteomics techniques for analysis of protein modifications**

| **Stability Proteomics Method** | **Application** | **Description** | **Advantages** | | **Limitations** | | **Publications** |
| --- | --- | --- | --- | --- | --- | --- | --- |
| Thermal Proteome Profiling (TPP) | PTM-TPP | TPP to examine the effects of post-translational modification on protein/proteome stability | | - Modification specific enrichment improves sensitivity - Enables a greater depth of PTM specific coverage | | - Has been shown to have issues with reproducibility - Cannot observe the effects of PTMs on totally unphosphorylated proteins | - Phosphorylation ^1^ - O-GlcNAcylation^2^ |
|  | Mutant TPP | TPP to examine the effects of protein mutation on protein/proteome stability | | - Detects changes in protein stability as a result of mutation - Detects changes in proteome thermal stability as a result of mutation such as those associated with protein-protein interactions | | - Cannot observe the effect of naturally expressed mutation - Cannot distinguish the effect of multiple, simultaneous mutations | - Temperature sensitive mutants^3, 4^ - Screening phosphor-mutants^5^ - Quantitation of low abundance proteoforms^433, 49, 117-11833, 48, 117-118^ |
| Limited Proteolysis (LiP) | PTM-LiP | LiP to examine the effect of PTMs on protein/proteome stability | | - Compatible with label-free quantitation | | - Some proteins may not be susceptible to proteolysis and may not be assayed | - Phosphorylation^6, 7^ |
| Stability of Proteins from Rates of Oxidation (SPROX) | PTM-SPROX | SPROX to examine the effect of PTMs on protein/proteome stability | | - Reversible chemical denaturation allows the calculation of thermodynamic constants - Covalent labelling allows analysis of site-specific unfolding | | - Methionine is a relatively rare amino acid and may not exist in a structurally relevant location in all proteins | - Phosphorylation^6^ |
